# Doxycycline inhibits electric field-induced migration of non-small cell lung cancer (NSCLC) cells

**DOI:** 10.1038/s41598-019-44505-8

**Published:** 2019-05-30

**Authors:** Hui-Fang Chang, Hung-Tien Cheng, Huai-Yi Chen, Wing Kiu Yeung, Ji-Yen Cheng

**Affiliations:** 1 Research Center for Applied Sciences, Academia Sinica, Taipei, Taiwan; 2 Department of Engineering and System Science, National Tsing Hua University, Hsinchu, Taiwan; 3 Nano Science and Technology Program, Taiwan International Graduate Program, Academia Sinica and National Tsing Hua University, Hsinchu, Taiwan; 4 Department of Mechanical and Mechatronic Engineering, National Taiwan Ocean University, Keelung, Taiwan; 5 Institute of Biophotonics, National Yang-Ming University, Taipei, Taiwan; 6 College of Engineering, Chang Gung Engineering, Taoyuan, Taiwan

**Keywords:** Lab-on-a-chip, Non-small-cell lung cancer

## Abstract

Adenocarcinoma, large cell carcinoma and squamous cell carcinoma are the most commonly diagnosed subtypes of non-small cell lung cancers (NSCLC). Numerous lung cancer cell types have exhibited electrotaxis under direct current electric fields (dcEF). Physiological electric fields (EF) play key roles in cancer cell migration. In this study, we investigated electrotaxis of NSCLC cells, including human large cell lung carcinoma NCI-H460 and human lung squamous cell carcinoma NCI-H520 cells. Non-cancerous MRC-5 lung fibroblasts were included as a control. After dcEF stimulation, NCI-H460 and NCI-H520 cells, which both exhibit epithelial-like morphology, migrated towards the cathode, while MRC-5 cells, which have fibroblast-like morphology, migrated towards the anode. The effect of doxycycline, a common antibiotic, on electrotaxis of MRC-5, NCI-H460 and NCI-H520 cells was examined. Doxycycline enhanced the tested cells’ motility but inhibited electrotaxis in the NSCLC cells without inhibiting non-cancerous MRC-5 cells. Based on our finding, further *in-vivo* studies could be devised to investigate the metastasis inhibition effect of doxycycline in an organism level.

## Introduction

Over the past few decades, lung cancer has been the most common malignancy and the most common cause of cancer-related deaths globally^1^. Lung cancers are classified as small cell lung cancers (SCLC) and non-small cell lung cancers (NSCLC), both of which exhibit independent growth and spreading behaviour. NSCLC accounts for approximately 85% of all lung cancer cases; it usually develops and spreads less rapidly than SCLC^2^. NSCLC has three major subtypes: (1) adenocarcinoma, which is usually found in the outer parts of the lung, (2) large cell carcinoma, which can develop in any part of the lung and (3) squamous cell carcinoma, which is usually found in the middle airways of the lungs^3^. Numerous patients with NSCLC die within the first few years of diagnosis, and the five-year survival rate is low^4^. Cancer metastasis is the major cause of cancer morbidity and mortality, accounting for about 90% of cancer deaths^5^. Cancer cell migration and invasion are initial steps in metastasis^6^.

Previous studies have reported considerable transepithelial potentials in lumens of living organisms^7,8^. Electrotaxis, or galvanotaxis, is the directional migration of adherent cells towards the cathode or the anode under direct current electric field (dcEF)^9,10^. Electrical fields (EFs) play key roles in physiological activities, such as cell division^11^, differentiation^12^, migration^13^, and death^14^. Some lung cancer cell types exhibit electrotaxis in applied dcEFs. For example, human lung adenocarcinoma A-549 cells migrated to the cathode in dcEFs^15^, while human lung adenocarcinoma CL1-5 cells migrated towards the anode. However, human lung adenocarcinoma CL1-0 cells, which are less malignant than CL1-5^16^, do not exhibit observable electrotactic responses^9^. Considerable evidence has demonstrated that cancer cells undergo reorientation and migration directionally under EF, which has potential implications for metastasis^17–19^. A previous study demonstrated that EF in the tumour micro-environment could play a critical role in lung cancer metastasis by guiding cell migration^20^. Therefore, EF may represent a key guidance cue and trigger for directional migration of cancer cells, in turn facilitating invasion and metastasis.

Doxycycline is an antibiotic widely used to treat bacterial infections^21–23^. Numerous studies have reported that doxycycline exhibits anti-tumour and pro-apoptotic activity. Furthermore, it decreases the tumour burden, as indicated by clinical trials, as a chemotherapeutic agent for several cancers^24–27^. Fife *et al*. demonstrated doxycycline inhibit human breast cancer cell (MDA-MB-435) migration, diminishes breast cancer cell lines’ proliferation, decreases their gelatinolytic (MMP) activity^24^, inhibits proliferation and induces apoptosis in human osteosarcoma cells^25^, and induces apoptosis in human breast cancer cells and prostate cancer cells *in vitro*^26^. In addition, Duivenvoorden *et al*. reported that doxycycline could be useful in the treatment of osteoblastic bone metastasis^27^. Several studies have also reported that doxycycline inhibits cancer metastasis^28–30^. In a study by Lokeshwar *et al*., doxycycline inhibited cell proliferation, invasion, and metastasis in prostate cancer^28^ while Qin *et al*. demonstrated doxycycline suppressed NCI-H446 lung cancer cell proliferation and metastasis^29^. Zhong *et al*. further demonstrated that doxycycline inhibits breast cancer metastasis^30^. Literature reports have demonstrated doxycycline’s anti-metastasis effects, thus examining the role of doxycycline in NSCLC could help exploit its anti-cancer potential.

This study’s aim is to demonstrate that doxycycline inhibits EF-induced migration of NSCLC cells. We investigated the migration responses of human fetal lung fibroblast cell line MRC-5, human large cell lung carcinoma cell line NCI-H460 (H460) and human lung squamous cell carcinoma cell line NCI-H520 (H520) to external direct current electric fields (dcEFs). This work is a two-fold study on NSCLC migration, where we explore the influence of (1) EF and (2) doxycycline on the electrotaxis of MRC-5, H460 and H520 cells.

## Results and Discussions

### The electrotaxis of MRC-5, H460 and H520 cells

**Figure 1:**
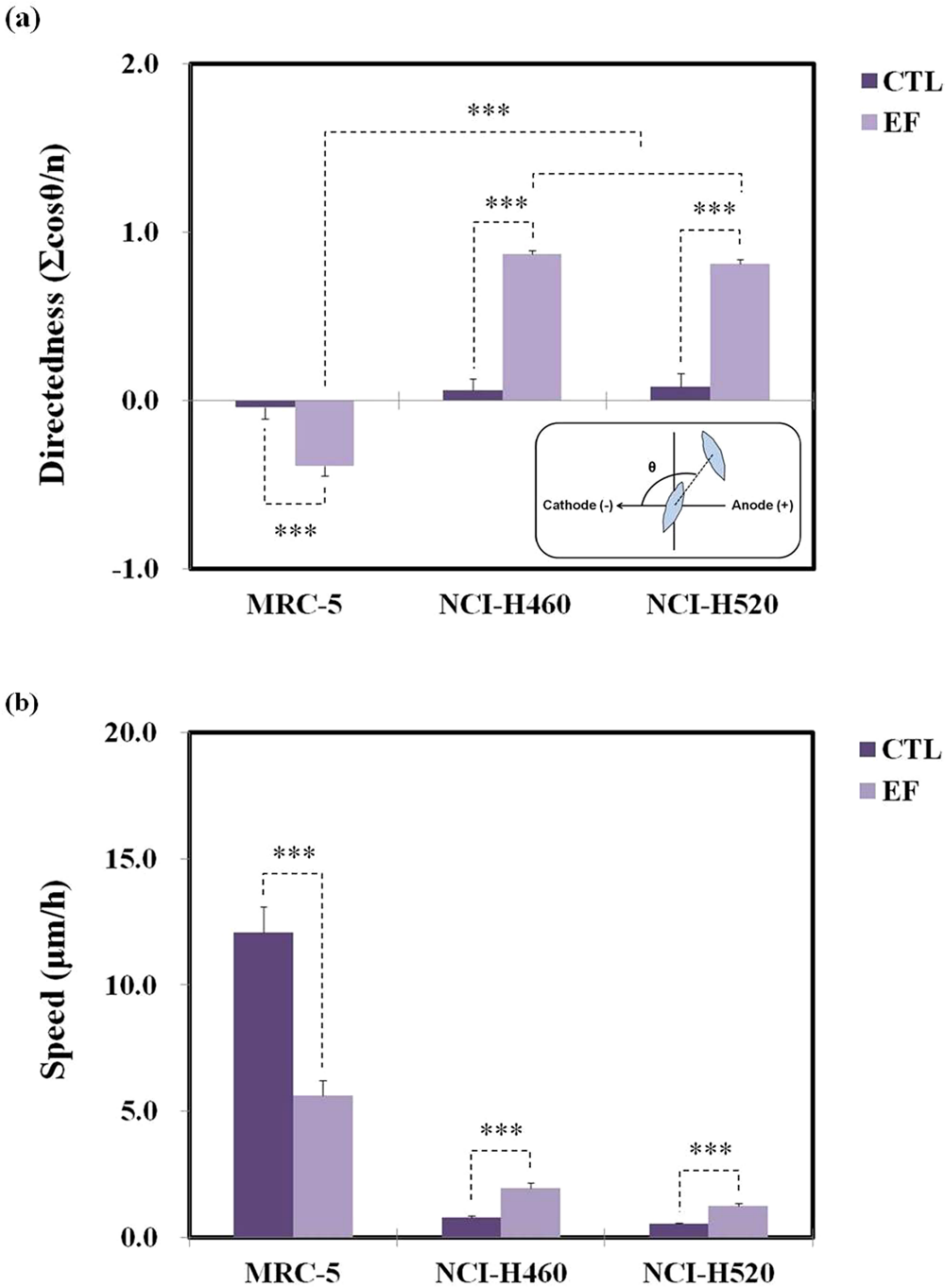
The (**a**) directedness and (**b**) migration speed of MRC-5, H460 and, H520 cells without (control [CTL]) and with dcEF (300 mV/mm) stimulation for 6 h. The inset in (A) shows the definition of the directedness. Each bar in the figure was calculated from data of 30 cells in three independent experiments. The EF is from the right to the left.

Under dcEF stimulation, MRC-5, H460 and H520 cells exhibit considerable electrotaxis. Our data (Fig. 1a) showed that MRC-5 cells migrated towards the anode and H460 and H520 cells migrated towards the cathode under 300 mV/mm. The directedness (defined in ‘Image analysis and data analysis’) of the MRC-5, H460 and H520 were −0.39 ± 0.06, 0.87 ± 0.02 and 0.81 ± 0.03 under the dcEF stimulation for 6 h, respectively. In brief, there were significant differences between the directedness in MRC-5, H460 and H520, and in the CTL group under the dcEF over 6 h.

The endpoint migration speeds (hereafter referred to as migration speed) of MRC-5, H460 and H520 cells, with and without the dcEF for 6 h, are presented in Fig. 1b. The migration speed of the MRC-5 cells without dcEF stimulation for 6 h were 12.08 ± 1.02 μm/h, while the migration speeds decreased to 5.61 ± 0.59 μm/h under dcEF stimulation for 6 h. The migration speeds of the H460 cells in the CTL group were 0.79 ± 0.05 μm/h without dcEF stimulation for over 6 h and increased to 1.96 ± 0.20 μm/h under dcEF stimulation. Similarly, the migration speeds of the H520 cells in the CTL group were 0.53 ± 0.05 μm/h while those under dcEF stimulation increased to 1.24 ± 0.09 μm/h for 6 h. Overall, the migration speeds of H460 and H520 cells under the dcEF stimulation were higher compared to those of the CTL group. Conversely, MRC-5 cells migration speeds under dcEF stimulation were lower than those in the CTL group. Therefore, EF stimulation enhanced the directional migration speed of NSCLC cells but decreased that of non-cancerous cells.

Several studies have shown that different types of cells migrate under EF^9,15,18,31,32^. Huang *et al*. observed that lung adenocarcinoma CL1-5 cells migrate towards the anode under a mV/mm dcEF of 300^9^. Pu *et al*. observed that human breast cancer DA-MB-231 cells and rat mammary adenocarcinoma MTLn3 cells migrated towards the anode when 50–400 mV/mm dcEFs were applied^31^. Conversely (Similarly but in the opposite direction), Djamgoz *et al*. observed that rat prostate cancer MAT-LyLu cells moved towards the cathode under a 300 mV/mm dcEF^18^. In addition, Tsai *et al*. observed that oral squamous cell carcinoma HSC-3 cells migrated towards the cathode under dcEF stimulation^32^ while Yan *et al*. observed migration of human lung adenocarcinoma A549 cells towards the cathode under a 300 mV/mm dcEF^15^. Irrespective of the observed electrotaxis direction, almost all cancer cells exhibit enhanced directional migration under externally applied EF.

In summary, the human large cell lung carcinoma H460 cells and human lung squamous cell carcinoma H520 cells migrated towards the cathode under a dcEF strength of 300 mV/mm. In contrast, human fetal lung fibroblast MRC-5 cells migrated towards the anode. In addition, the migration speed of H460 and H520 cells under dcEF stimulation was higher than in the CTL group. In previous studies, cancer cells with higher metastatic potential have exhibited stronger electrotactic responses^9,15,18,31,33^. In the present study, H460 and H520 NSCLC cells also exhibited strong electrotactic responses. Considerable transepithelial potentials exist in living organisms^7,8^. EF’s enhancement of cancer cells migration could also potentially occur *in vivo*. An in-depth study on the influence of electrotaxis on different lung cancer cell could potentially enhance the understanding of EF-induced NSCLC migration.

### Electrotaxis in non-small cell lung cancers (NSCLC)

To better understand the electrotaxis in NSCLC, quantitative analysis of electrotaxis in A-549, CL1-0, CL1-5, MRC-5, H460 and H520 cells under dcEF stimulation for 2 h were compared in Table 1. Under dcEF stimulation, CL1-5 NSCLC cells^9^ and MRC-5 cells migrate towards the anode; while A549^15^, H460 and H520 NSCLC cells migrate towards the cathode. Previous studies show that cell morphological changes are critical to cell migration. Furthermore, it has been indicated that cell morphology also plays a vital role in embryonic development, wound healing and cancer spread^34^. In addition, *in vitro* studies have demonstrated that the presence of endogenous or an exogenous EF is another factor that controls cell morphology and guides cell migration^17,35–37^. Notably, cell morphology analysis revealed that the cells migrating towards the cathode under dcEF stimulation exhibited epithelial-like morphology (A549^38–40^, H460^40–42^ and H520^43^). Conversely, CL1-5^44–46^ and MRC-5^47–49^ cells, which migrated towards the anode, exhibited fibroblast-like morphology. The difference suggests that cell morphology could indicate the directedness during electrotaxis.

**Table 1:** Electrotaxis in A-549, CL1-0, CL1-5, MRC-5, H460 and H520 cells with dcEF stimulation for 2 h.

Morphology	Cell name	Cell type	EF (mV/mm)	Directedness ± SEM
Fibroblast-like	MRC-5	Human fetal lung fibroblast cells	300	−0.32 ± 0.07
Fibroblast-like	CL1-5^9^	Human lung adenocarcinoma cell	375	−0.60 ± 0.04
Epithelial-like	CL1-0^9^	Human lung adenocarcinoma cell	375	−0.01 ± 0.07
Epithelial-like	A-549^15^	Human lung adenocarcinoma cell	300	0.76 ± 0.12
Epithelial-like	NCI-H460	Human lung large cell carcinoma	300	0.80 ± 0.03
Epithelial-like	NCI-H520	Human lung squamous cell carcinoma	300	0.50 ± 0.06

Expression of signalling molecules is reportedly involved in the electrotactic response mechanism. Previous studies have explored the mechanism of electrotaxis in lung adenocarcinoma cells^15,33,50,51^. Yan *et al*. observed that human lung adenocarcinoma A549 cells exhibited epidermal growth factor receptor (EGFR) dependent electrotaxis responses. Treatment of A549 cells with EGFR inhibitors, AG1478, and cetuximab reduced the enhancement of phosphorylated ERK (pERK) and phosphorylated AKT (pAKT). This suggested EGFRs play a key role in electrotaxis of A549 cells^15^. However, it has also been demonstrated that electrotaxis in CL 1-5 cells is EGFR independent^51^. In addition, applying EF to CL1-5 cells leads to up-regulation of ACVR1B (activin A receptor type 1B) and CTTN (cortactin) genes, and down-regulation of phosphatase and tensin homolog (PTEN)^33^. In addition, Akt and ribosomal protein S6 (RPS6) phosphorylation increased in CL 1-5 cells under dcEF stimulation. Although CL 1-0 and CL1-5 cells share the same origin, decreased RPS6 phosphorylation was observed in CL 1-0 cells under EF stimulation^51^. The findings suggest expression of different signalling molecules in the NSCLC cells’ electrotactic responses. However, the signalling pathway in MRC-5, H460 and H520 cells under EF remains unclear. Further investigation of the roles of signalling molecules in MRC-5, H460 and H520 cells electrotaxis would lead to better understanding of electrotaxis in NSCLC. Here, we began by chemically inhibiting the migration of the cells studied.

### Effect of doxycycline on MRC-5, H460 and H520 cell viability

**Figure 2:**
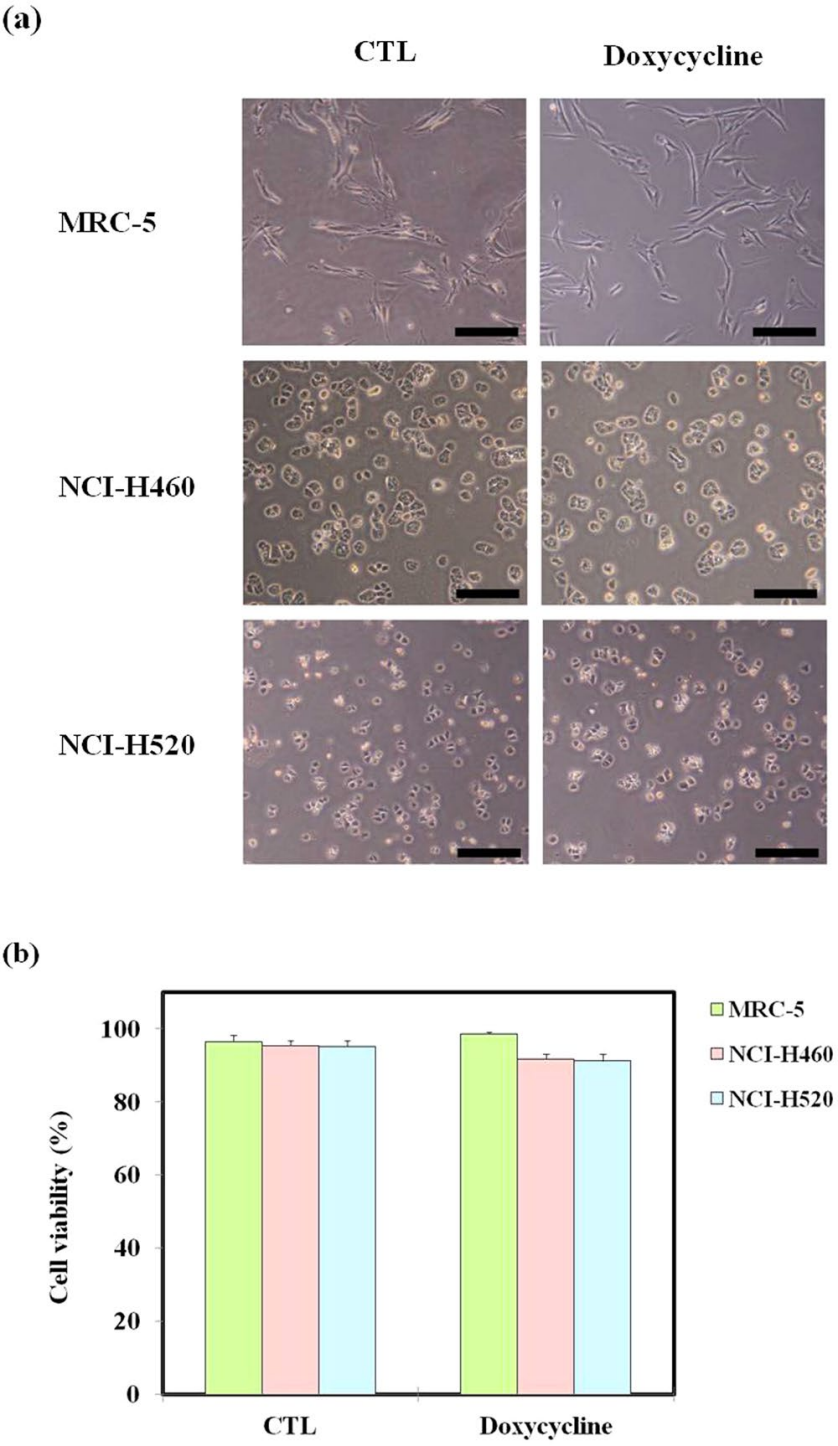
The effect of doxycycline on the cell viability of MRC-5, H460 and H520 cells was not significant. (**a**) Cell morphology of MRC-5, H460 and H520 cells treated without and with doxycycline after 24 h. There were no observable changes. Scale bars: 100 μm. (**b**) Cell viability of the CTL and the doxycycline-treated group after 24 h. Doxycycline concentration: 10 μg/mL.

Cancer cell migration and invasion are the initial steps during metastasis^6^. Previous studies have reported increased extracellular matrix degradation and matrix metalloproteinase (MMP) activity, which facilitate tumour spread^26,52^. In addition, doxycycline is a nonspecific MMP inhibitor. Before studying doxycycline’s effect on electrotaxis, we verified that doxycycline exhibited no cytotoxicity. This was achieved by determining cell viability in the presence of doxycycline. According to a previous study, A549 cells incubated with 10 μg/mL doxycycline exhibited approximately 90% cell viability^53^. In the present study, the cells were grown using a cell culture chip, with and without doxycycline, at the same concentration. Cell morphology in the CTL and the doxycycline-treated groups after 24 h are presented in Fig. 2a. The morphology of MRC-5, H460 and H520 cells exhibited no significant changes and good viability in both the CTL and the doxycycline-treated groups. As shown in Fig. 2b, both the CTL and the doxycycline-treated groups showed viability at approximately 91%, indicating good cell growth inside the chip. Based on the finding, doxycycline’s effect on MRC-5, H460 and H520 cells electrotaxis were investigated as described below.

### The effect of doxycycline on electrotaxis of MRC-5, H460 and H520 cells

**Figure 3:**
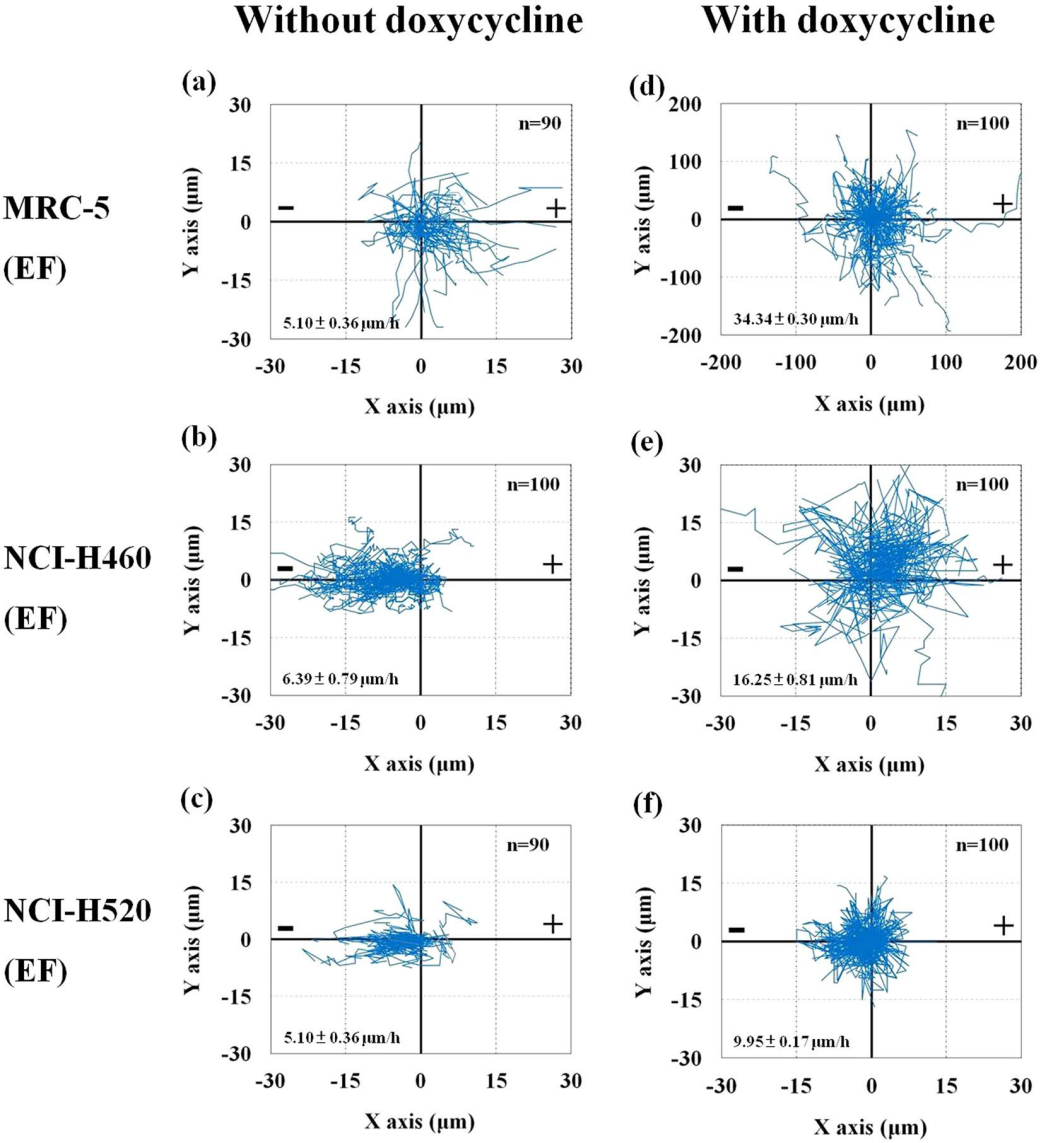
The cell trajectory of (**a**,**d**) MRC-5, (**b**,**e**) H460 and (**c**,**f**) H520 cells treated without (**a**–**c**) and with (**d**–**f**) doxycycline in the electrical field (EF) groups. The cell positions are normalised as if all begin at the origin (X = Y = 0).

Previous studies have reported that the MMP pathway plays a role A549 and CL1-5 lung adenocarcinoma cells’ migration^29,54^. Qin *et al*. reported that doxycycline inhibited A549 lung adenocarcinoma cells migration and invasion by inhibiting MMP activity^29^. In the present study, the MMP pathway’s role in cell electrotaxis was investigated using doxycycline. The cell trajectories of doxycycline-treated MRC-5 (Dox-MRC5), H460 (Dox-H460) and H520 (Dox-H520) cells with dcEF stimulation are shown in Fig. 3. By comparing the cell trajectories of the Dox-MRC5-EF to the MRC5-EF groups, the cells with doxycycline exhibited less preferential migration towards the anode. In addition, Dox-H460-EF and Dox-H520-EF cell groups also showed hindered and reduced directedness, respectively.

Quantitative analysis of trajectory speed could be carried out using the time-lapsed trajectory plots illustrated in Fig. 3. The results indicated that adding doxycycline increases motility significantly (2 to 6 fold). The changes in electrotaxis directedness in H460 and H520 cells were not because of retarded motility. Our result suggested that, in the cells, the signalling pathways’ effects, with regard to the determination of the directions and on the cellular motion, ought to be different.

**Figure 4:**
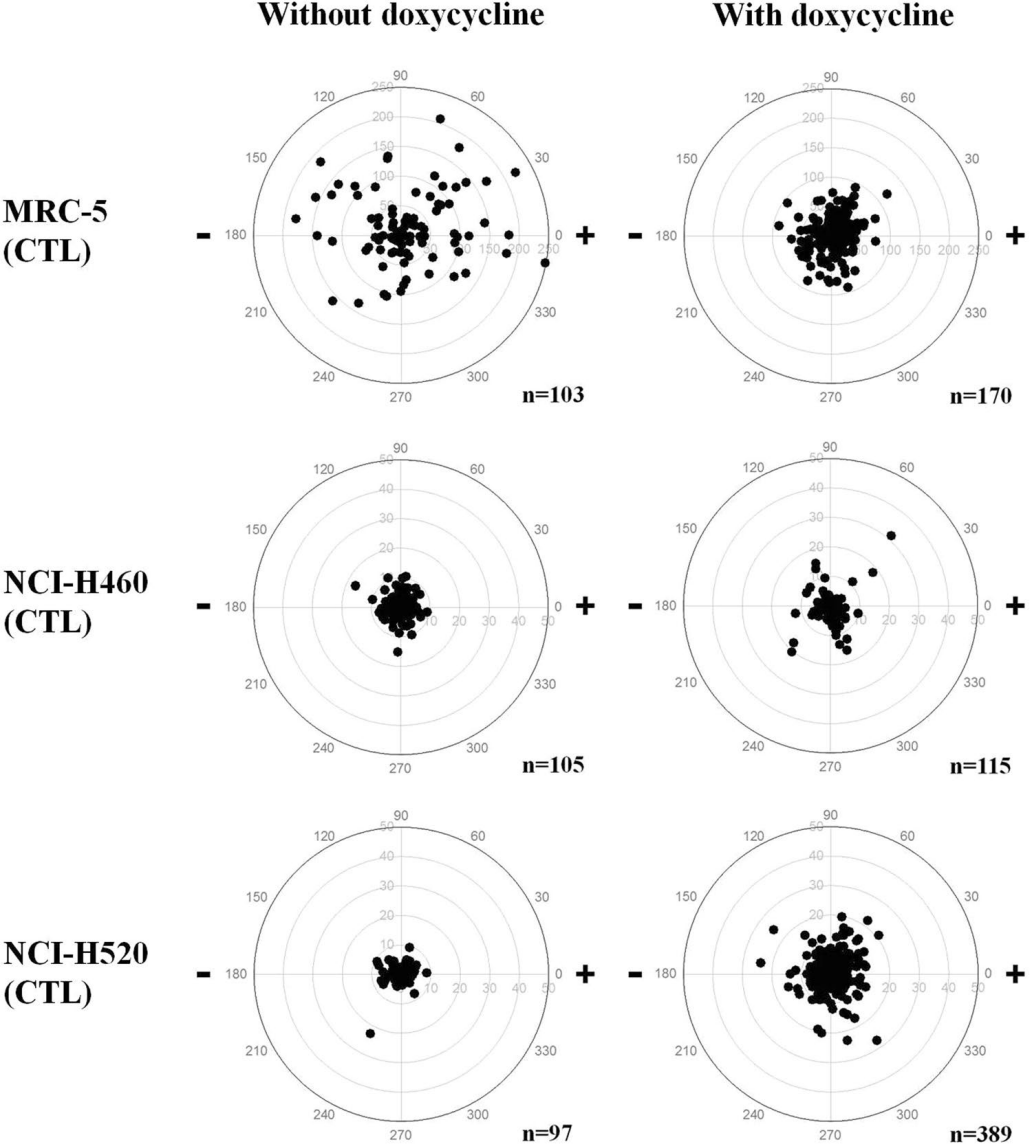
The polar plots of the cell migration of MRC-5, H460 and H520 cells treated with and without doxycycline in the control (CTL) groups. The EF stimulation was treated for 6 h.

**Figure 5:**
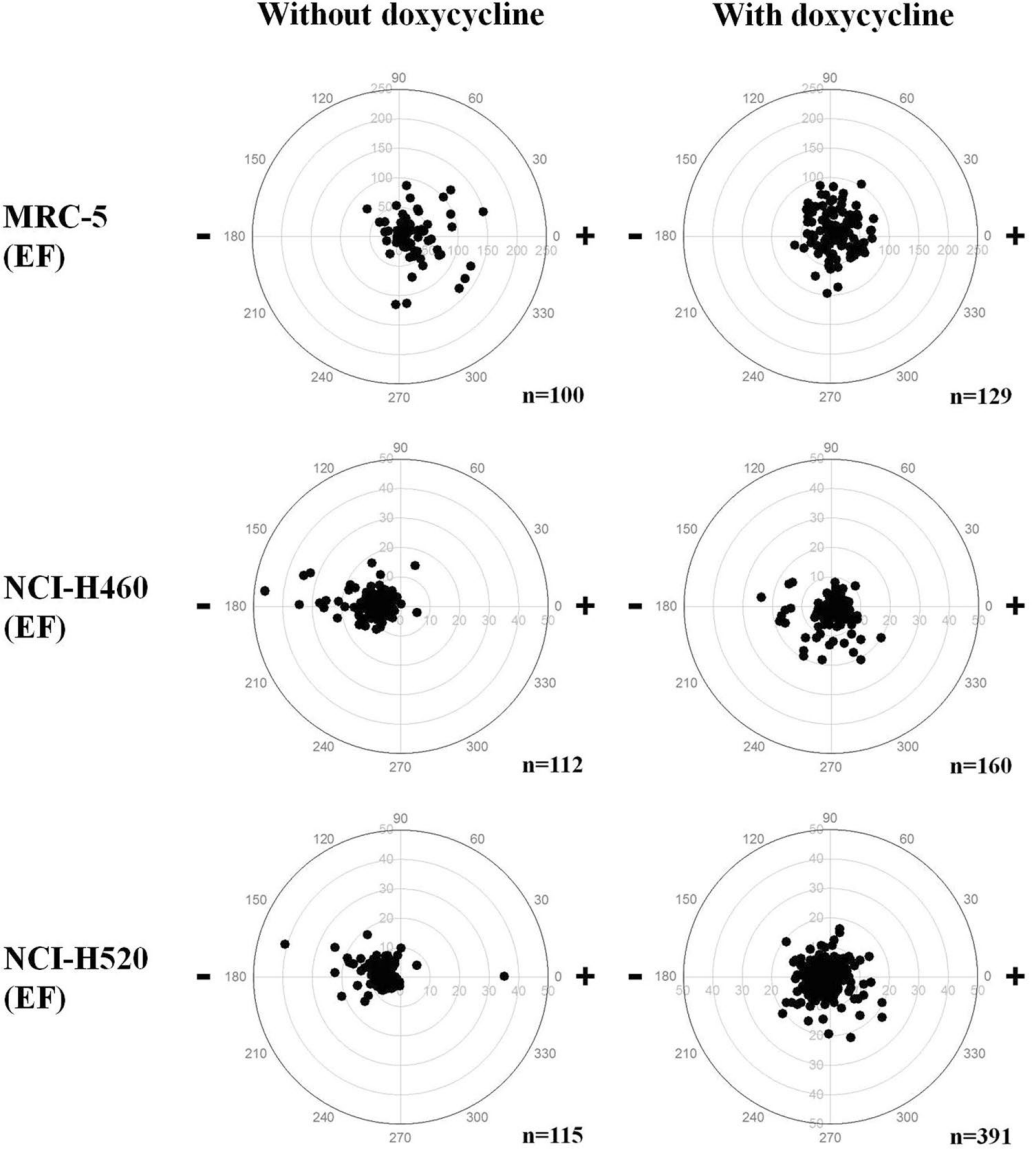
The polar plots of the cell migration of MRC-5, H460 and H520 cells treated with and without doxycycline in the electrical field (EF) groups. The EF stimulation was treated for 6 h.

In the present study, the cell migration of Dox-MRC5, Dox-H460 and Dox-H520 cells, with and without dcEF, for 6 h are depicted as polar plots in Figs 4 and 5. Detailed quantitative and statistical analyses of the electrotaxis of the MRC-5, H460 and H520 cells for 6 h in EF are presented in Table 2. The data showed that directedness in H460 cells was significantly different between the H460-EF and the Dox-H460-EF groups. Similarly, directedness in H520 cells was significantly different in both the H520-EF and Dox-H520-EF groups. Doxycycline eliminates cathodal migration in Dox-H460-EF compared to H460-EF groups. In comparison, doxycycline only partially reduces cathodal migration in Dox-H520-EF groups compared to H520-EF groups. The findings confirmed that doxycycline affects electrotaxis in H460 and H520 but not in MRC-5 cells. Previous studies have reported that doxycycline inhibits migration of human MDA-MB-435 breast adenocarcinoma cells^24^, NCI-H446 human small cell lung cancer cells^29^, and MCF-7 and MDA-MB-231 human breast carcinoma cells^30^. Our findings suggest doxycycline may regulate tumour progression by inhibiting electrotaxis.

**Table 2:** The electrotaxis in MRC-5, H460 and H520 cells without and with doxycycline.

Groups	Cell number	Directedness (Σcosθ/n) ± SEM			Migration speed (μm/h) ± SEM		
		6 h	*p* ^a^	*p* ^b^	6 h	*p* ^a^	*p* ^b^
MRC5-CTL	103	−0.04 ± 0.07	***		12.08 ± 1.02	**	
MRC5-EF	100	−0.39 ± 0.06	***	^—^	5.61 ± 0.59	**	^—^
H460-CTL	105	0.06 ± 0.07	***		0.79 ± 0.05	***	
H460-EF	112	0.87 ± 0.02	***	***	1.96 ± 0.20	***	***
H520-CTL	97	0.08 ± 0.08	***		0.53 ± 0.05	***	
H520-EF	115	0.81 ± 0.03	***	***	1.24 ± 0.09	***	***
Dox-MRC5-CTL	161	−0.09 ± 0.05	^—^		5.88 ± 0.29	^—^	
Dox-MRC5-EF	122	−0.24 ± 0.06	^—^	^—^	6.08 ± 0.35	^—^	^—^
Dox-H460-CTL	112	−0.10 ± 0.05	^—^		0.78 ± 0.07	^—^	
Dox-H460-EF	160	−0.05 ± 0.06	^—^	***	0.90 ± 0.06	^—^	***
Dox-H520-CTL	389	0.08 ± 0.04	***		0.75 ± 0.04	^—^	
Dox-H520-EF	391	0.27 ± 0.04	***	***	0.76 ± 0.03	^—^	***

*p*^a^, *p* value of independent t-test between CTL and EF.

*p*^b^, *p* value of independent t-test between EF and Dox-EF.

^—^, no significant; *p < 0.05; **p < 0.01; ***p < 0.001. SEM, standard error of the mean.

Signalling molecules play key roles in cancer cell progression and metastasis and influence cancer cells’ migratory properties^5^. Previous studies reported using doxycycline as a broad inhibitor for multiple MMPs^55–57^. Several reports support MMPs’ roles in tumour progression, including growth^58^, angiogenesis^59^, invasion^60^, and migration^61^. Therefore, decreasing MMP activity could inhibit cancer cell invasion and metastasis^54,62–64^. Electrical stimulation, combined with ionising radiation, synergistically suppresses migration and invasion of human glioblastoma multiforme cells based on MMP-9 inhibition^65^. Therefore, the differences in electrotactic response between H460 and H520 cells could be explained by different levels of MMP expression in the cells, despite their similarity in cell morphology. Studies based on doxycycline’s inhibition of electrotaxis could be devised to understand the electrotaxis mechanism better. However, there are limited studies of MMP expression of NSCLC under the influence of doxycycline and electrotaxis. Further investigation is required for the expression of MMP under doxycycline with/without EF stimulation could be measured.

In this study, we demonstrate that EF, in combination with doxycycline treatment, results in antagonism. Doxycycline inhibited EF-induced directional migration in H460 and H520 cells. The inhibition is potentially not attributable to retarded cellular motion since doxycycline increased cellular motility. This study provides insights that could facilitate understanding doxycycline’s role in lung tumour progression and potential regulation of NSCLC electrotaxis of NSCLC.

## Conclusion

Previous studies reported that human lung adenocarcinoma CL1-5 cells migrate towards the anode^9^, whereas the human lung adenocarcinoma A549 cells migrate towards the cathode^15^. In the present study, we investigated electrotaxis in H460 and H520 NSCLC cells. As a control, non-cancerous MRC-5 lung fibroblasts were included in our study. Our results showed that H460 and H520 cells migrated towards the cathode under a dcEF strength of 300 mV/mm, but MRC-5 cells migrated towards the anode. H460, H520 and MRC-5 cells have epithelial-like morphology and fibroblast-like morphology, respectively. Therefore, the cells exhibited different electrotactic responses. In addition, the migration speed of H460 and H520 cells increased under dcEF stimulation. We demonstrated that doxycycline increased motility but inhibited cathodal migration in H460 and H520 cells. The findings confirmed that doxycycline-regulated electrotaxis in NSCLC. Based on our finding, further *in-vivo* studies could be devised to investigate the metastasis inhibition effect of doxycycline in an organism level.

## Methods

### Fabrication of optically-transparent electrotactic chip

**Figure 6:**
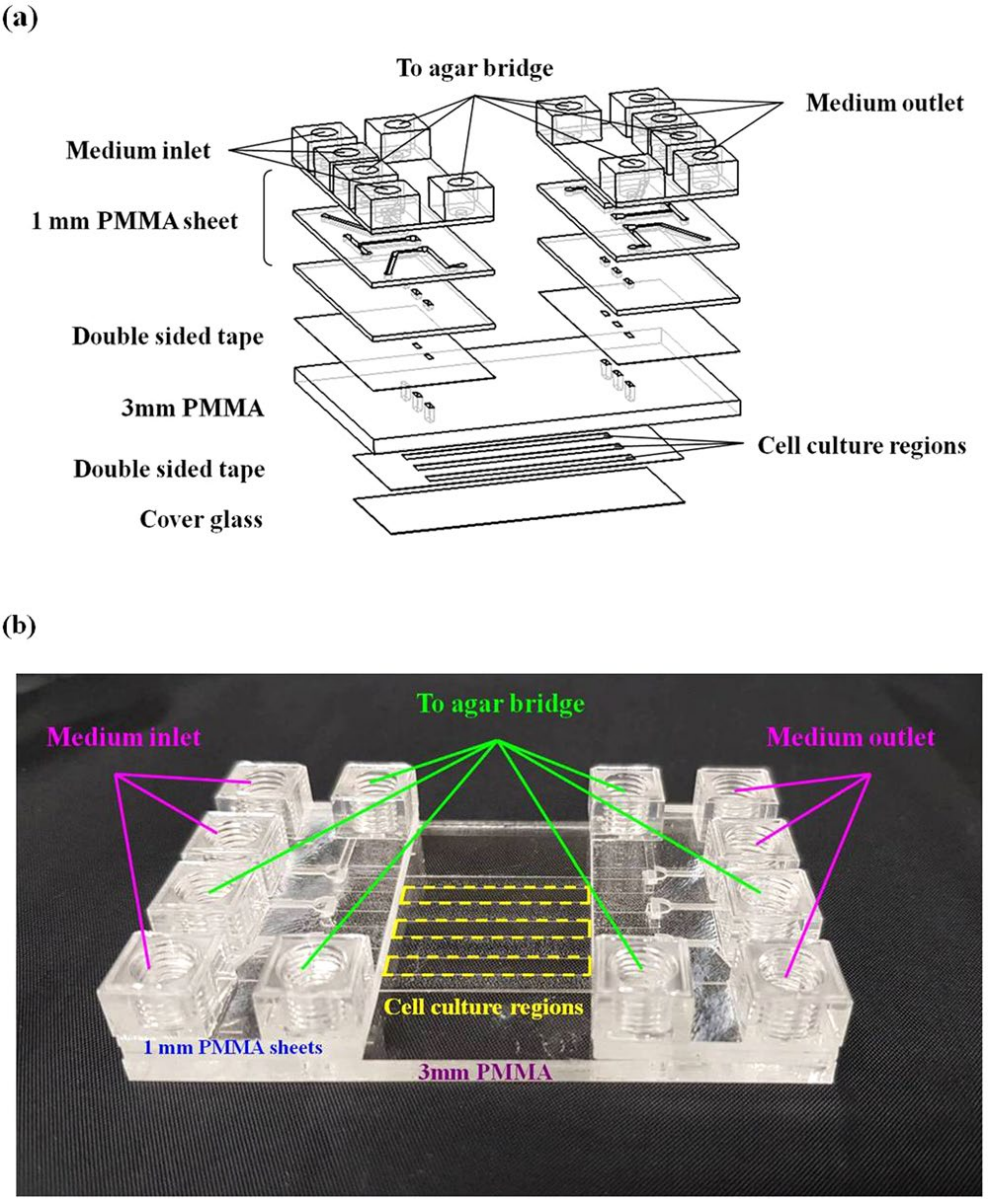
The detailed configuration of the electrotactic chip. (**a**) The optically-transparent electrotactic chip assembly design. The chip has connecting holes for the medium inlet and outlet and the agar salt bridges. The cells were cultured in the cell culture regions. The width, length and thickness of the cell culture region were 3 mm, 42 mm and 70 μm, respectively. (**b**) Photograph of the electrotactic chip.

The optically**-**transparent electrotactic chip configuration is illustrated in Fig. 6. The detailed fabrication procedure has been described in our previous works^9,12,32,33,50,51^. The electrotactic chip was designed to perform three independent electrotaxis experiments simultaneously. There were three sets of connections for medium inlet/outlet and agar bridges. From the top to the bottom, the chip was composed of three 1 mm PMMA sheets, a 70-μm-thick polyester double-sided tape (PET 8018; 3M, St. Paul, MN), a 3 mm optical grade PMMA sheet (ACRYPOLY^®^ PMMA Sheet; CHI MEI Corporation, Tainan, Taiwan), a 70-μm-thick polyester double-sided tape and a cover glass (BB024060A1 Deckgläser; Thermo Fisher Scientific Gerhard Menzel, Braunschweig, Germany). The double-sided tape’s biocompatibility was confirmed in our previous study^66^. In brief, the patterns on the polymethyl methacrylate (PMMA) sheets and the double-sided tape were drawn using AutoCAD software (Autodesk, San Rafael, CA). The patterns were fabricated using a CO_2_ laser scriber (ILS-II; LTT Group, Hsin Chu City, Taiwan). All the layers’ components were disinfected using UV irradiation for 30 min before assembling the chip. To obtain a bubble-free channel during long-term cell culturing, the chip was put in a vacuum chamber for 30 min.

This chip had high optical transparency and good durability. The novel chip allowed the carrying out of three series of cell stimulation studies simultaneously. In addition, the chips were designed to be suitable for confocal microscopic examinations. The chips could also be used for investigating the effects of doxycycline with and without dcEF stimulation simultaneously in a single experiment.

### The system used for electrotaxis study

**Figure 7:**
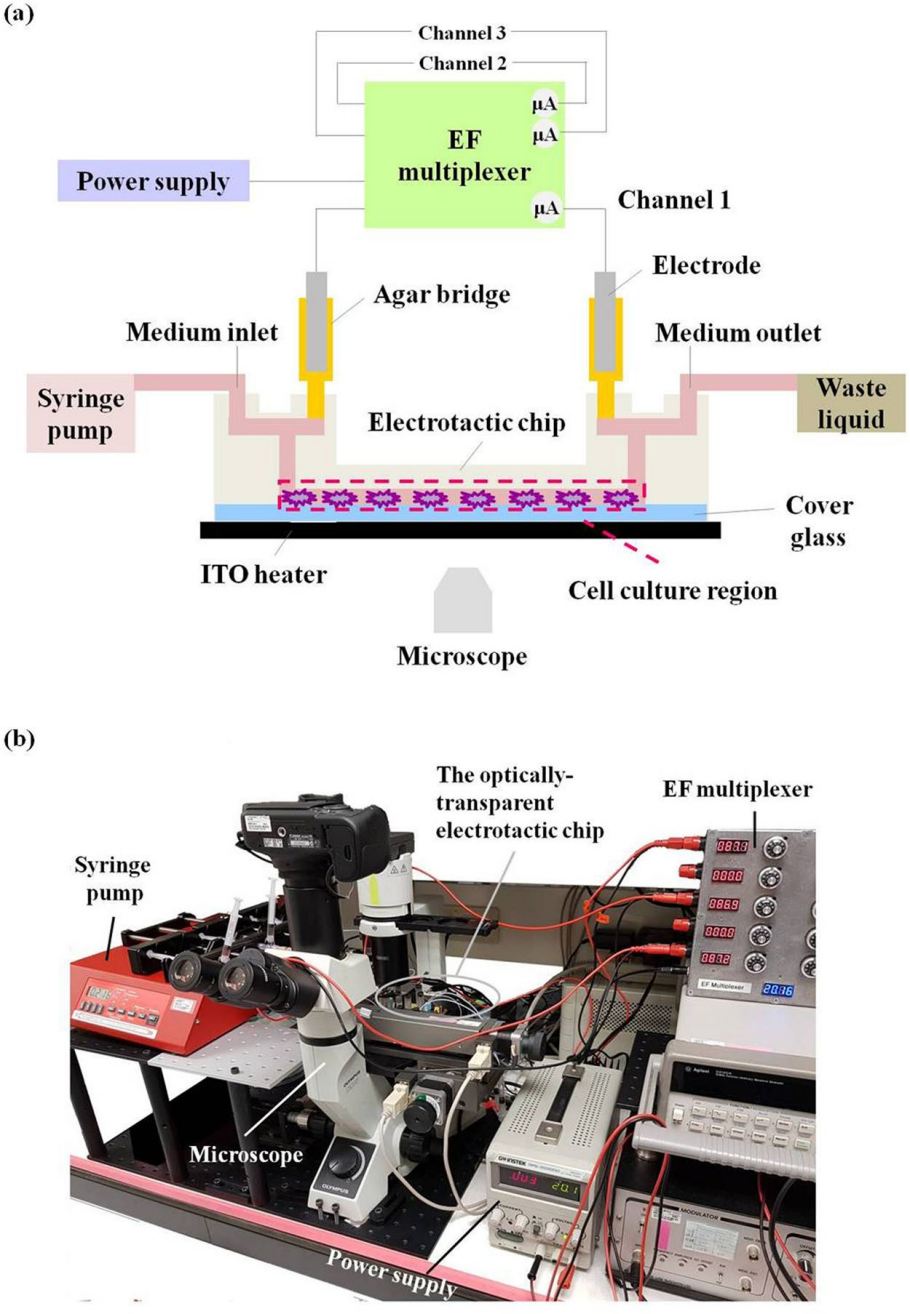
The system used for the electrotaxis study. (**a**) The configuration of the entire system for the electrotaxis study. (**b**) A photo picture showing the setup on a laboratory bench.

The system configuration is illustrated in Fig. 7. The entire system is built onto an inverted phase contrast microscope (CKX41; Olympus, Center Valley, PA) equipped with a digital camera (60D; Canon, Japan) to monitor cell migration within the cell culture region in the chip. The chip is placed onto a transparent indium–tin–oxide heater (ITO glass, part no. 300739; Merck, Whitehouse Station, NJ) that is locked on a programmable X-Y-Z motorised stage (Tanlian Inc., Taiwan). The ITO surface temperature is controlled by a proportional–integral–derivative (PID) controller (TTM-J4-R-AB; Toho Electronics, Nagoya, Japan) and maintained at 37 °C. An additional K-type thermocouple (TPK-02A; Tecpel, Taipei, Taiwan) is clamped between the chip and the ITO heater to monitor the temperature of the cell culture regions within the chip. Ag/AgCl electrodes are inserted in the 1.5% agar salt bridges (Sigma-Aldrich, USA) as the electrical connections to the cell culture medium. In this setup, the Ag/AgCl electrodes provide a stable pH and current during the electrotaxis experiment^32^. The medium inlet is connected to a syringe and a syringe pump (NE-1000; New Era Systems Inc., Farmingdale, NY). An in-house designed EF multiplexer is connected to a DC power supply (GPS-3030DQ; GW Instek, Taiwan). This novel multiplexer design facilitates independent and precise control over current flow in each cell culturing region. The EF multiplexer is a circuit that includes the culture chamber in the circuit and connects all the chambers in an electronic network. The function of the EF multiplexer is to provide different EF strengths to different chambers in the chip. The concept is depicted in Supplemental Figure S1.

### Cell culture and maintenance

The human fetal lung fibroblast cell line MRC-5, human large cell lung carcinoma cell line NCI-H460 (H460) and human lung squamous cell carcinoma cell line NCI-H520 (H520) were obtained from Bioresource Collection and Research Center (BCRC) in Taiwan. BCRC has performed short tandem repeat polymerase chain reaction (STR-PCR) profile and confirmed the cells’ identity.

MRC-5 cells were cultured in 90% Eagle’s minimum essential medium supplemented with 10% fetal bovine serum (FBS), 0.1 mM non-essential amino acids and 1.0 mM sodium pyruvate. H460 and H520 cells were cultured in 90% RPMI 1640 medium with 10% fetal bovine serum. All media and reagents for cell culture were purchased from GIBCO (Thermo Fisher Scientific Inc., USA).

The cells were incubated in tissue culture polystyrene (TCPS) flasks (Nunc, Roskilde, Denmark), placed in an incubator filled with 5% CO_2_ atmosphere and maintained at 37 °C. Cells were subcultured every 3–4 days. The cells used in the present study were within 10 to 15 passages. The cultured cells were routinely tested for mycoplasma using a commercial PCR kit (e-Myco plus, iNtRON Biotech, Korea). All the cells used in the present study were free of mycoplasma contamination.

### Electrotaxis experiment

The syringes for introducing cells and medium were connected to the inlet of the electrotactic chip through 3-way stopcocks (NIPRO, Japan), Teflon tubing and fittings (IDEX Corp., USA). The chip was sequentially flushed from inlet to the outlet with PBS. The chip was then filled with a culture medium supplemented with 10% FBS and was ready for cell seeding.

To prepare cells for electrotaxis, cultured cells were washed by PBS and then trypsinized from TCPS flask. The trypsin was removed by centrifugation and cells were re-suspended in the complete medium. The cells (1 × 10^6^ cells/ml) were infused by manual pumping into the chip via the medium outlet. The cells were cultured in the chip for 4 h so they would adhere to the cover glass. After adherence, fresh medium was pumped through the cell culture region, via the medium inlet, at a flow rate of 20 μl/h using a syringe pump. The cells were then cultured in the chip for an additional 20 h to allow cell growth.

Subsequently, cells were pretreated with the complete medium with and without 10 μg/mL doxycycline for 24 h on the chip. Afterwards, the EF was then applied, and the electrotaxis experiment was carried out. During the electrotaxis experiment, the medium flow was stopped to avoid the effect of liquid flow on cell migration.

For applying EF in the electrotactic chip, the chip was first connected to the agar salt bridges. The electrical circuit was completed by connecting the DC power supply to the Ag/AgCl electrodes on the chip (Supplemental Figure S2). Typically, a voltage of 20 V was applied on the electrodes to obtain EF strength of 300 mV/mm in the cell culture regions of the electrotactic chip. The electric current flowing through the electrotactic chamber was continuously monitored by the ammeter connected serially with the chip. For the control group, the experimental setup was the same except that the EF strength was 0 mV/mm.

### Cell viability assay

The cell viability was measured using SYTOX^®^ Green/Hoechst 33342 staining (Molecular Probes, Thermo Fisher Scientific, Eugene, OR). SYTOX^®^ Green is a high-affinity, nucleic acid stain that penetrates cells with compromised plasma membranes without penetrating the live cell membranes^67^.

The effect of doxycycline on cell viability was studied by treating cells with 10 μg/mL doxycycline for 24 h. After treatment, SYTOX^®^ Green (1 μM) and Hoechst 33342 (16.2 μM) was pumped into the chip at a flow rate of 100 μl/min for 10 min. Cells were then incubated at 37 °C for 20 min. The cells were then examined using a confocal fluorescence microscope (TCS SP5; Leica Microsystems, Wetzlar, Germany).

### Image analysis and data processing

Time-lapse images from the phase contrast microscope were analysed using Wimasis WimTaxis (Wimasis GmbH Munich, Germany). WimTaxis is a tool for quantitatively evaluating migration and is suitable for analysing time-lapse videos from phase contrast techniques. In each experimental condition, over 90 cells from three independent experiments were analysed. Cell migration parameters were analysed as follows:

1. Length of cell migration: distance (in μm) from the starting point to the final position of the cell’s centroid.
2. Migration rate: the average distance of cell migration per hour.
3. Directedness: defined as average cosine θ, Σcosθ/n, where θ is the angle between the vector of the applied EF (from positive to negative) and the vector from the starting point of a cell to its final position; n is the number of cells used for analysis. The directedness is −1 for a cell moving towards the anode, and +1 for a cell moving towards the cathode. For a group of randomly migrating cells, the directedness is 0^18^.
4. Trajectory speed: total trajectory distance divided by the total time, where the trajectory distance is total accumulated length of the track.

For cell viability analysis, the integrated fluorescence intensity of SYTOX^®^-stained cells (λ_Ex_ = 488 nm, λ_Em_ = 523 nm) and Hoechst-stained cells (λ_Ex_ = 350 nm, λ_Em_ = 460 nm) were calculated. Cell viability was then determined as cell viability in percent = 100% − (SYTOX^®^ intensity/Hoechst intensity) × 100%.

All data were expressed as mean ± SEM. Statistical significance was determined using Student’s t-test. *p* < 0.05 represents statistical significance. The asterisk (*) denotes *p* < 0.05, double asterisks (**) denote *p* < 0.01 and triple asterisks (***) denote *p* < 0.001.

## Supplementary information

Supplementary FigS1

Supplementary FigS2
