# Supplementary figures and images for: Doxycycline inhibits electric field-induced migration of non-small cell lung cancer (NSCLC) cells
Source: Sci Rep. 2019 May 30;9:8094. doi: 10.1038/s41598-019-44505-8 (PMC6542854; doi:10.1038/s41598-019-44505-8)

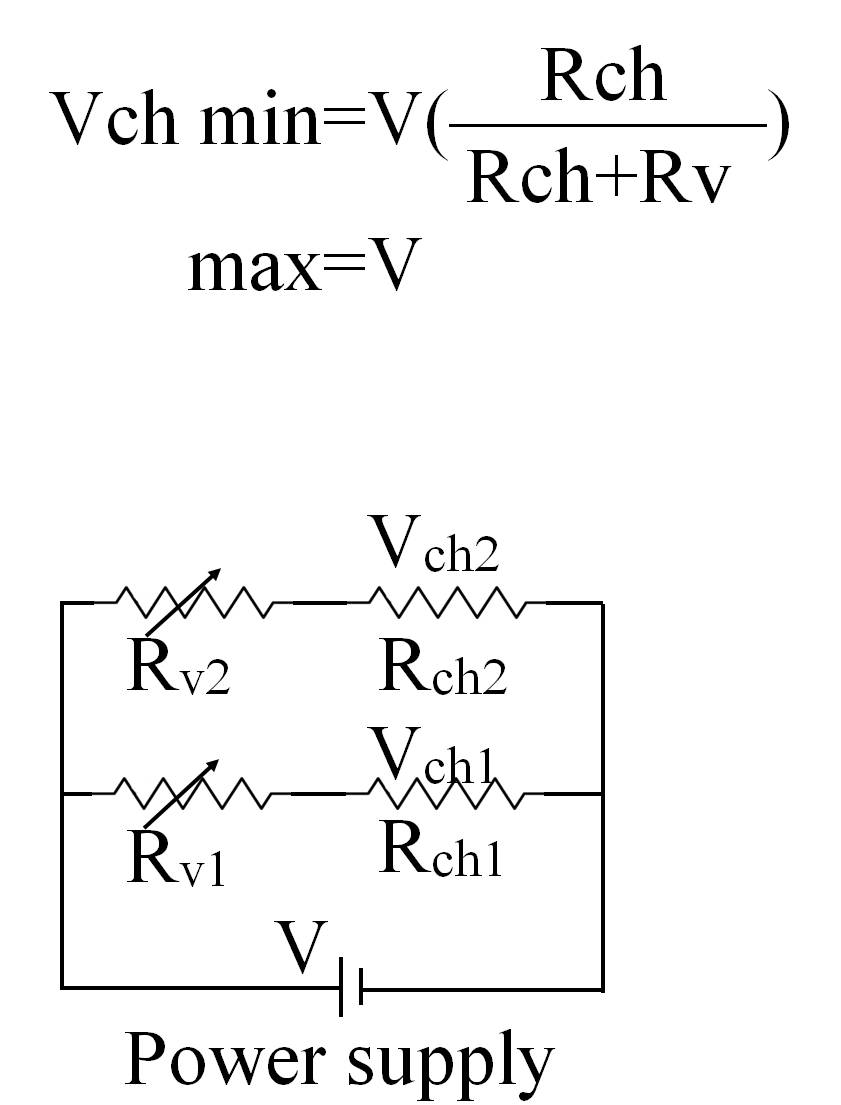

Supplement: Supplementary file 1 — Supplementary FigS1 [file 41598_2019_44505_MOESM1_ESM.jpg]

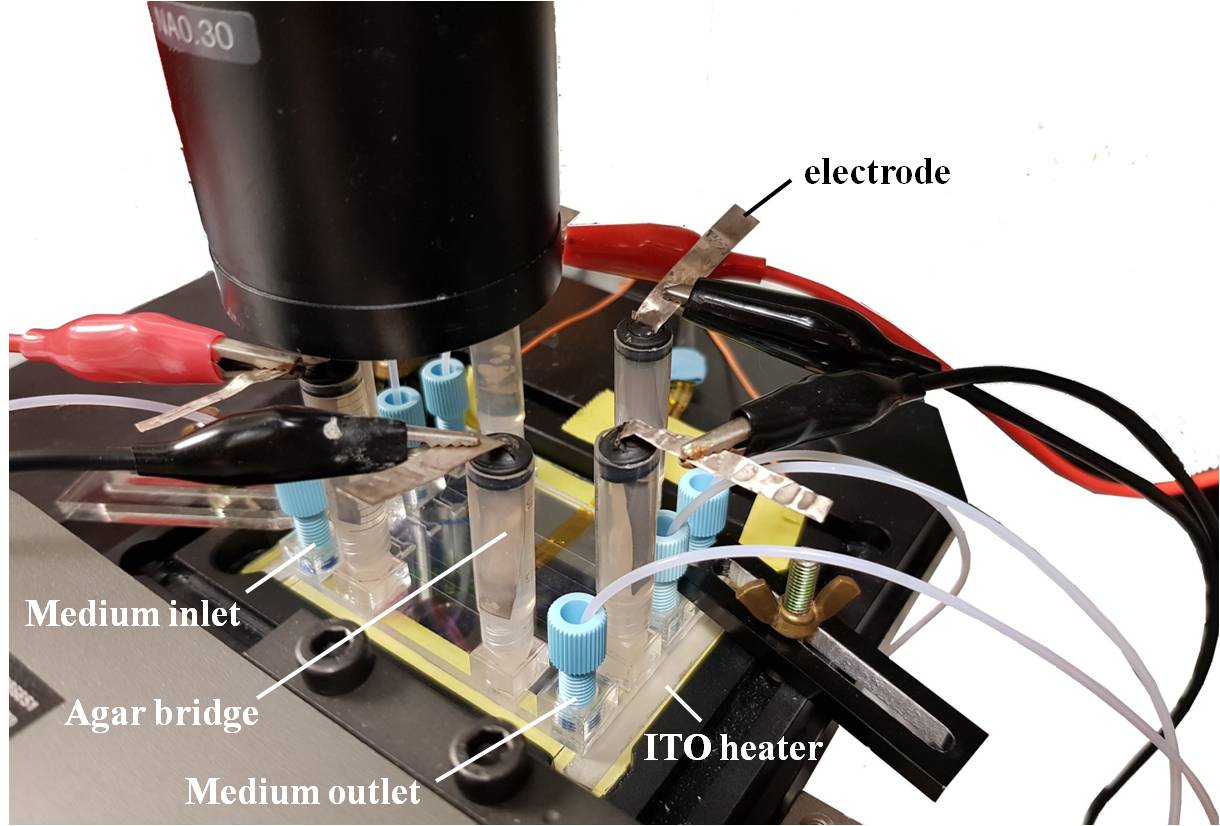

Supplement: Supplementary file 2 — Supplementary FigS2 [file 41598_2019_44505_MOESM2_ESM.jpg]
